# Perceived Value Similarity With Important Others: Well-Being Implications for Emerging Adults

**DOI:** 10.3389/fpsyg.2022.716952

**Published:** 2022-05-30

**Authors:** Jelisaveta Belic, Mandy Boehnke, Klaus Boehnke

**Affiliations:** ^1^Bremen International Graduate School of Social Sciences (BIGSSS), Jacobs University Bremen, Bremen, Germany; ^2^Bremen International Graduate School of Social Sciences (BIGSSS), Universität Bremen, Bremen, Germany

**Keywords:** values, value similarity, emerging adults, well-being, important others

## Abstract

Emerging adults establish, question, and reestablish their values within the most diverse social contexts. Every social context privileges expressing certain values and/or punishes expressing conflicting ones. This makes a similarity between one’s own values and those preferred in one’s life contexts psychologically desirable (person–environment fit). This study focuses on the similarity of individuals’ values with the perceived values of important others from five immediate social contexts, namely, family, friends, intimate partner, study group, and work group, and their relationship with life satisfaction. The sample consisted of emerging adults from Serbia interacting with the five mentioned contexts (*N* = 479). A mobile app with a game-like survey was launched to collect the data. The data indicated a positive association between life satisfaction and perceived value similarity with one’s family and with one’s intimate partner. Value similarity with friends and study and work colleagues emerged as insignificant. Identity centrality and the general importance of the immediate social contexts were studied as possible moderators. Identity centrality showed no moderation effect, whereas general importance of the intimate partner did: High importance of the intimate partner *decreased* the positive effect of value similarity on well-being.

## Introduction

To indicate the importance of values, it is worth emphasizing that for decades, psychology, as well as sociology and political science, have been providing different high-impact theories to conceptualize values, both at the personal level and the societal level (Inglehart, 1971; Hofstede and Bond, 1984; Huntington, 1996; Rokeach, 2008). We used one of the most prominent models in psychology, Schwartz’s original theoretical model of ten personal values organized in a circumplex, indicating the dynamics between values. Each value in this model is defined by its motivational goal, which is assumed to guide our actions. Values are conceptualized as being organized hierarchically, thus marked as priorities, indicating the importance of each of the values to the individual (Schwartz, 1992).

Until now, researchers have mainly been interested in looking into either adolescents’ or adults’ values. Adolescence was chosen since according to the seminal life-cycle model of Erikson that phase was—at the time when the model was proposed—recognized as the developmental period characterized by identity crises and by value formation (Weiland, 1993; Scott and Scott, 1998). Adults were mostly studied without concern for their specific age (Boehnke et al., 1994). However, in light of worldwide changes resulting in the noticeable postponement of crucial steppingstones into adulthood in industrialized societies (i.e., family foundation and/or job commitment), Arnett coined *emerging adulthood* as the term for a new developmental period that young adults go through (Arnett, 2000). In light of the shakiness of achieving the criteria to be considered as adults, emerging adults’ identity together with their values go through a moratorium phase. Studies have indicated that the psychological well-being of this population is being challenged, thereby drawing attention to the central research question of this study (Kessler et al., 2007; Hunt and Eisenberg, 2010).

While occupied with establishing their values as an important part of their identities, emerging adults’ lives are embedded in a multiplicity of social contexts. Our quasi-axiomatic assumption is that having value preferences similar to the value preferences prevailing in one’s social context is desirable and associated with positive subjective well-being (Sagiv and Schwartz, 2000). This has been confirmed repeatedly across diverse contexts such as family, study group, and work group (e.g., Bengtson, 1975; Sagiv and Schwartz, 2000; Pereyra-Rojas et al., 2017). However, there is little research including more than one social context (e.g., Boehnke et al., 2007; Hadjar et al., 2012) at a time. The existence of multiple relevant social contexts through which individuals interact with their important others is usually ignored. In this situation, we propose to simultaneously investigate the relationship between subjective well-being and perceived value similarity in *five* immediate social contexts. We chose perceived, or “subjective,” value similarity because of the following: with regards to subjective well-being the belief of isolation/connection is more important than the “objective” value congruence (reported by significant others). For more information, please refer to Boehnke’s Journal of Cross-Cultural Psychology (2001) article—the section on the difference between perceived and objective, as well as to Wolf et al. (2020).

In a nutshell, following the pertinent literature, we chose the five most relevant social contexts for emerging adults, consisting of their important others (family, friends, study and work colleagues, and intimate partners). We used a short form of the Schwartz value questionnaire (Schwartz, 2003) to assess the value preferences of the participants as well as to ask for the perceived value preferences among members of the mentioned social contexts. To assess subjective well-being, we used the well-established measure of its cognitive aspect, Diener et al.’s (1985) Satisfaction With Life Scale. We propose that perceived value similarity with intimacy groups (i.e., family, friends, and intimate partners) contributes to subjective well-being to a greater extent than perceived similarity with task groups (i.e., work and study colleagues). Furthermore, based on the previous studies (Sagiv and Schwartz, 2000; Roccas and Brewer, 2002; Daniel et al., 2012), we assume identity centrality—in simple terms, how important the different life contexts are for a given individual’s identity as well as how important in general these contexts are—to function as moderators. The more important the social context is, the stronger the connection between perceived value similarity and satisfaction with life is assumed to be.

**FIGURE 1:**
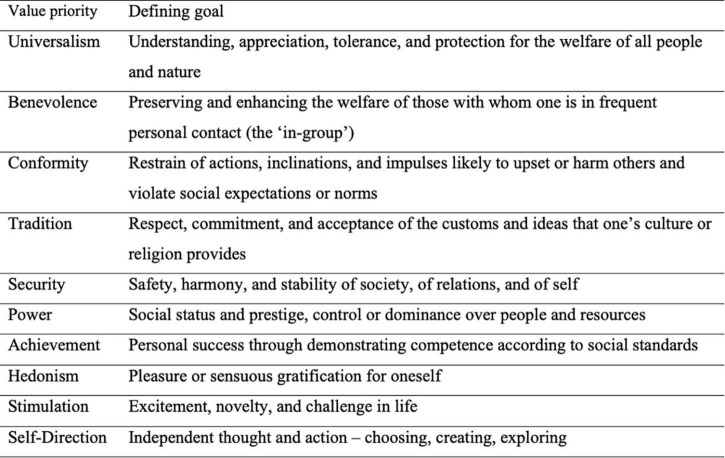
Value priorities and their defining goals (adapted from Schwartz, 1992).

Since this study is concerned with individuals (not aggregates like groups, societies, or even continents as in Huntington’s work), it was decided to adopt the empirically established and cross-culturally supported way to conceptualize personal values, Schwartz’s (1992, 1994) theory of basic values, as its frame of reference. Schwartz defined values as abstract beliefs directing our action (Kluckhohn, 1951; Schwartz, 1992; Rokeach, 2008). According to the theory, these vital beliefs—personal values—are linked to affect; people feel happy when the goals driven by their values are achieved, sad when their values are threatened or need to be protected, etc. Personal values differ in their importance, and for each individual, they are organized hierarchically as priorities. Data from cross-cultural studies support the idea that personal values, as conceptualized by Schwartz, are structured circularly, indicating the compatibilities and incompatibilities between different value priorities (Borg et al., 2017). Each value priority feeds into unique, desirable goals and thereby guides action, which in turn simultaneously comes at the expense of other, conflicting, values and goals behind them. Schwartz (1992) distinguished ten value types: self-direction, stimulation, hedonism, achievement, power, security, conformity, tradition, benevolence, and universalism, each representing different motivational goals (Figure 1)^1^.

**FIGURE 2:**
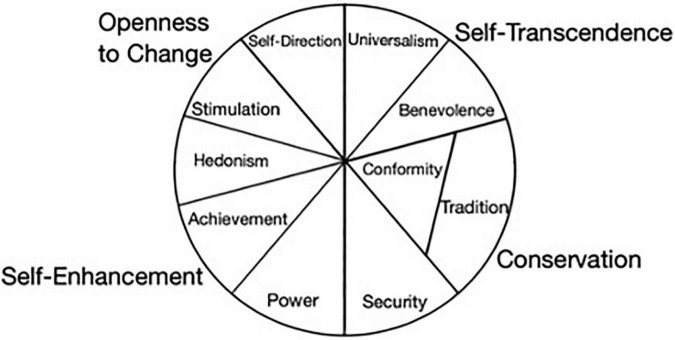
Relationship among value priorities (adopted from Schwartz, 1992).

Relations among values are specified by the circumplex presented below (Figure 2). Values close to each other in the circle are similar to each other, while values on the opposite poles are conflicting, creating two higher-order bipolar dimensions: openness to change vs. conservation and self-transcendence vs. self-enhancement.

Until now, value researchers typically were not overly interested in the developmental aspects of value formation (see, however, Boer and Boehnke, 2015). They usually focused on investigating values among adults, not distinguishing emerging from mid or late adulthood, while lately there has been work by Borg (2019) that filled the gap to some extent. We followed Borg’s work by offering input on value preferences and their relationship to well-being for an age group that has recently drawn increasing attention, namely, emerging adults.

In recent decades, most Western countries have seen major changes in the transition to adulthood, which is usually described as a series of transition events, including completion of school education, entry into the labor market, leaving the parental home, establishing a first durable partnership, and entering parenthood (Buchmann and Kriesi, 2011). It has been argued that changes in the transition to adulthood have led to an increasing destandardization and increased complexity of the transition to adulthood (Cohen et al., 2003; Shanahan et al., 2005).

Arnett (2000) first proposed the term emerging adulthood to map a newly arisen developmental period fitting to the realities of 18–29 years of age living in industrialized societies. Young people of this age increasingly tend not to settle romantically or job-wise, unlike their peers only a decade earlier. They rather choose longer education or training paths (Arnett, 2000, 2007, 2011), and—not to forget—need no longer to formally start a family to engage in sexual activities. Focusing on the self, they search for meaningful lives (Mayseless and Keren, 2014). To achieve that, they explore possibilities, interact with universities and the labor market, and engage in exchanges with people and groups both online and offline (Arnett, 2007; Vannucci et al., 2017). Major changes such as globalization and increasing digitization and with it an expansion of reality increased choices and opportunities. Due to the absence of long-standing commitments to the tasks considered as the milestones of adulthood (family foundation and job inception), identity lingers unstable and open for shaping, but at the same time open to the feeling of “in-between” (Arnett, 2007). A topic related to what sociologists call individualization, which describes the phenomenon that individuals, freed from traditional roles and structures, increasingly have the freedom and the duty to shape their own lives and construct their biography (e.g., Beck and Beck-Gernsheim, 2001).

For a long time, identity formation had been seen as a developmental task of adolescence (Weiland, 1993). However, identity issues are still quite noticeable in emerging adulthood (Schwartz et al., 2005); identity formation clearly has not been achieved fully by the end of adolescence. Values are considered to play a central role in personal identity (Brewer and Roccas, 2001; Verplanken and Holland, 2002), and even though a 14-year-old is already equipped with the cognitive apparatus to subscribe to a differentiated set of personal values, adolescents and later on emerging adults seemingly go through an increasingly more difficult process of questioning and reestablishing their personal values to achieve the status of an adult and form their identity (Arnett, 1997). These identity processes are happening inside a multiplicity of social contexts *via* social roles individuals occupy (Gergen and Gergen, 1997). With a diversified social surrounding, tracking and evaluating the process of transmission of values will not be simple and predictable. As for emerging adults, combined with a great variety of experiences offered in a globalized world, the attempts to deal with instability often encompass negotiations with close others that are used as a compass in finding their own identity. Potentially, this might lead to psychological challenges and deteriorating subjective well-being (Arnett, 2007). A comprehensive World Health Organization (WHO) study conducted across seventeen countries found substance use, mood disorders, and certain anxiety disorders (i.e., generalized anxiety and panic disorder) to have their onset and peak in the twenties (Kessler et al., 2007; Whiteford et al., 2013). Furthermore, this life period is an especially sensitive time for the feeling of loneliness (Baker, 2012; Asher and Weeks, 2014), attempted to be overcome by the use of the Internet, social media, and smartphone applications such as Tinder (Hood et al., 2017; Sumter et al., 2017). Loneliness refers to social isolation and the lack of congruence between an emerging adult and their respective social context can contribute to that feeling (Sagiv and Schwartz, 2000). Thus, there are important reasons to investigate emerging adults’ values, which are likely to be related to life satisfaction and psychological well-being.

There is a long tradition of researchers trying to position particular values as contributing to or endangering personal mental health. In these studies, self-direction, benevolence, and universalism were found to be weakly to moderately positively correlated with mental health (Strupp, 1980; Jensen and Bergin, 1988). However, studies by Boehnke et al. (1998) or by Sagiv and Schwartz (2000) suggested that we need to shift our focus from particular values *per se* to the interaction of values a person holds with the social environment they inhabit. This interaction alone seems to influence people’s subjective well-being. Sagiv and Schwartz (2000) embedded this finding into the person–environment fit hypothesis: People are more probable to reach higher levels of well-being when their value priorities are congruent with value priorities prevailing in their environment. Three underlying mechanisms are offered for this assumption: environmental affordances, social sanctions, and within-person conflict (Sagiv and Schwartz, 2000). The first two are reflecting characteristics of a given social environment. First, contexts are providing opportunities for a person to express their values, making it beneficial and resource-efficient to exhibit value preferences in line with those prevailing in the context. Second, contexts are social interaction spaces and, thus, communicating values similar to those expressed by others creates satisfying social bonds whereas expressing different value preferences may lead to social sanctions. Finally, internal (within-person) conflict may become an issue because adolescents and emerging adults being brought up in close contact with the set of value preferences prevailing in their family, whereas during the individuation phase (Youniss and Smollar, 1987), a distancing from parental values becomes a developmental task (Hickel, 2013), which in turn may generate internal conflict.

However, there is no consensus in the literature about the social environments one should consider. Numerous studies confirm the person–environment value fit hypothesis for quite diverse, more and less abstract social contexts, e.g., culture and culture-related contexts (Musiol and Boehnke, 2013; Sortheix and Lönnqvist, 2014; Sortheix and Schwartz, 2017), workplace (Pereyra-Rojas et al., 2017), academic department (Sagiv and Schwartz, 2000), and family (Bengtson, 1975). Until now, value similarity mainly has been investigated in the context of value socialization or vertical transmission of values, from parents to their children (e.g., Boehnke, 2001; Boehnke et al., 2007; Barni et al., 2011). Although peers, as well as family, are recognized by researchers as the ones to offer alternative perspectives on appropriate values (Kuczynski et al., 1997), it is difficult to find studies on the similarity of value priorities between peers and their role in well-being. Furthermore, studies on this issue rarely include more than one social context at once. This is surprising since people interact with diverse social contexts that sometimes offer contradictory messages (Knafo and Schwartz, 2001).

The absence of consensus about the groups one should consider emerges because as such any social context could be seen as a potentially important social environment. Not only are the important socialization entities such as family, school, or peers relevant, but also are others that provide functions for work, leisure, or entertainment. When we think of social contexts, we can elicit many different criteria for their categorization (Ellemers et al., 2002): according to their function (expressive and instrumental, Umphress et al., 2003), or according to their entitativity (intimacy groups, task groups, social categories, and groups with loose associations, Lickel et al., 2000). Cooley (1909) introduced the concept of primary and secondary groups, primary groups such as the family of origin and close friends play an important role in the development of personal identity, whereas secondary groups such as coworkers or classmates are seen as less influential on one’s identity.

None of the studies reviewed so far differentiated social contexts when investigating person–environment fit. However, at least some of the immediate social contexts important for an individual, if not all, share value preferences: They typically belong to the same culture and are exposed to the same zeitgeist, i.e., mindsets characteristic for a given place and time (Boehnke et al., 2007). Consequentially, when looking, for example, into the study group or work group alone, one can find value similarities as impacting well-being, but if we complexify analysis with more social contexts, due to the possibility that some of them share values, this initial impact can be found to be attributed to value similarities of our participants with other social groups. Therefore, for this study, we chose not only social contexts consisting of significant others (such as family and intimate partners), which are theoretically assumed to be the main transmitters of values. Rather, to capture the diversity of social groups and test the aforementioned assumption, we chose *five* immediate social contexts consisting of persons considered to be important others in one’s life, namely, parents, intimate partner, friends, study, and work colleagues.

Finally, social contexts with their members differ based on their relevance for an individual, i.e., how important they are to a person. *Identity centrality*, as an indicator of the importance of a context for the individual, has been proposed to assess how crucial a part of one’s identity in different social contexts are. Identity centrality is believed to moderate the relationship between value preferences and well-being (Sagiv and Schwartz, 2000). Studies on migrant populations, for example, have revealed the importance of an individual’s identification with a social context and its members, in this case, the heritage in contrast to the settlement society (Chan and Tam, 2016). Sagiv and Schwartz (2000) suggested, furthermore, that a lack of congruence between personal value preferences and value preferences dominant in a social context is likely to be irrelevant to people’s well-being if a specific social context is not a component of a given individual’s identity or if there are other social contexts more relevant to their identity. Similarly, Dragolov and Boehnke (2015) suggested that an individual internalizes certain values depending on their compatibility with the worldview prevalent in a given country, assessed as the preference of specific social axioms (Leung and Bond, 2009). Since looking not only at the expressive, identity-related but also instrumental importance of given social contexts, we decided to also incorporate a comparative measure of the *general importance* of social contexts. In light of the reported findings, we assume the degree to which similarity between an individual’s and important others’ values affect well-being to be enhanced when the given social context is important, and potentially central to the identity of that specific individual. In other words, we assumed that the relationship between values and well-being is not simply determined by the degree of person–environment fit, but more so by the general importance and identity centrality of the social context.

This study investigates the interaction between personal values and the perceived values of important others and subjective well-being as an outcome, and whether the relationship is moderated by the importance (i.e., general importance and more specifically, identity centrality) of the five social contexts, which are family, intimate partner, friends, study, and work colleagues. Our first hypothesis draws on the simple person–environment fit hypothesis (Sagiv and Schwartz, 2000) and reads as follows: *Perceived value similarity of an individual with their respective social context will contribute to subjective well-being, regardless of the type of social context* (H1).

Furthermore, based on the characteristics of intimacy vs. task-based groups (Lickel et al., 2000), we assumed that the relationship between perceived value similarity and well-being will have a stronger effect for intimacy contexts (family, friends, and intimate partner) than for task-based groups (study and work contexts): *Perceived value similarity with family, friends, and intimate partner will affect subjective well-being more strongly than perceived value similarity with the study and work contexts* (H2).

**FIGURE 3:**
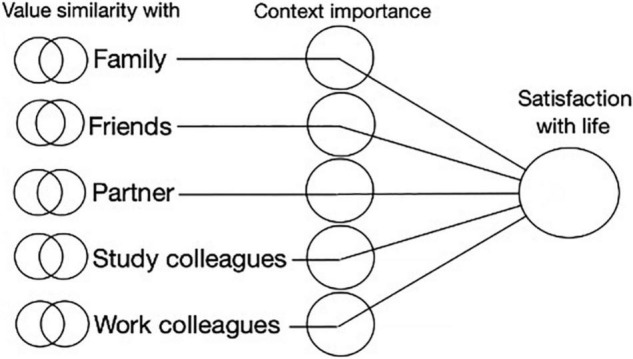
Model on the relationship between value similarity and life satisfaction moderated by context importance (general importance and identity centrality).

As elaborated, previous studies typically investigated the person–environment fit hypothesis only by including a single social context. However, in a real-life multiple-social-context setting, we are surrounded by various, often competing, values that different social groups encourage us to pursue. Hence, the relationship between personal values and subjective well-being becomes much more complex and multilayered than commonly addressed in pertinent research. To remedy this shortcoming, we tested the relationship between perceived value similarity and subjective well-being in five different contexts simultaneously (refer to Figure 3).

As a result, it became possible to test to what extent the relationship between value similarity and well-being is moderated by two context importance indicators, namely, (1) general importance and (2) specific identity-related importance of a given social context (i.e., identity centrality). As identity centrality is expected to play a moderating role (Sagiv and Schwartz, 2000), *we propose that perceived value similarity will contribute to subjective well-being depending on how important, and especially how identity central a social context is to the identity of an emerging adult, regardless of the type of social context. We expect that the more important the context is to a person, the more influential the implications of value similarity to subjective well-being outcome will be* (H3).

## Materials and Methods

### Procedure

Participants filled in the questionnaires in a game-like mobile app setting, on their phones, in their own privacy. For the piloting of the app, a small convenience sample had been cognitively interviewed (*n* = 4) and asked to rate different properties of the app (i.e., clarity of the language, instructions, interface, and moments of a more intense engagement or disengagement). These first pilot results informed further development of the app to secure a pleasant user experience, meant to facilitate obtaining answers as reliable and truthful/valid as possible. A preliminary version of the questionnaire was then given to another round of pilot participants (*n* = 10) to confirm that the product is ready to be launched. The application allowed users to answer the questions at their own pace, meaning they could stop when experiencing fatigue and continue where they stopped. In classical paper-pencil surveying, the probability is high that the repetitive nature of the instrument—questions on perceived value preferences had to be answered five times—will reduce data quality (Cape, 2010). The app paved the way for multiple questionnaires to be combined into a meaningful narrative of self-discovery, thereby ensuring that participants answer all questions and creating a game-like experience for its users. By filling out the questionnaires, participants were successfully completing levels in diverse visual surroundings accompanied by calming music on a quest to understand and assess well the diverse sets of psychological constructs relating to themselves and others, such as their values, their subjective well-being, and values of their social contexts. Deterding et al. (2011) showed that gamification of questionnaires is useful for increasing participants’ motivation. Users of our application were incentivized intrinsically, so to speak, to participate until the very end. Further information about the look of the app can be found on Open Science Framework (Belic, 2021). A screenshot of the original Serbian version of the response format and its English translation offered by the app is documented in Figure 6 towards the end of the article. The study was approved by the Ethics Committee (IRB Committee) of the Bremen International Graduate School of Social Sciences, to which all authors are affiliated.

### Participants

A snowball convenience sample of emerging adults from urban Serbia, predominantly Belgrade, was drawn for the study. Participants were contacted in different ways; some of them were reached through facilitators employed to assist with the project. Others were solicited through universities and companies offering part-time and so-called mini-jobs, which are the dominant job opportunity for emerging adults in Serbia. Finally, some of them were contacted directly through social media networks.

Initially, Arnett had proposed 18–25 years of age to be the range for this developmental period (Arnett, 2000) and later expanded it to people between 18 and 29 years of age (Arnett, 2011). The age range must be seen as rather flexible because what constitutes emerging adulthood largely depends on the specific cultural context, which dictates the pace of achieving independence criteria to a major extent (Negru, 2012). Bearing in mind that Serbia typically provides young people with conditions that impede a smooth path to independence (Tomanović and Ignjatović, 2006), and due to a lack of studies specifying the appropriate age range for emerging adulthood in this country, we initially allowed for participants between 18 and 40 years of age to participate *but then excluded those who had already achieved adulthood*, based on the following three independence criteria: (1) having finished education, (2) being in full-time employment, *and* (3) being married. The mean age of our participants was 24.63 (SD 4.22). As a comparison of the impact of *all five* social contexts was the central goal of our study, for analysis, we only included in our analyses those participants who had no missing data on any of the five targeted social contexts from the initial 997. Eventually, data from 479 Serbian emerging adults constituted the sample for this study, all of whom are students, with the gender distribution skewed toward female participants (355 out of 479).

### Instruments

Schwartz’s 11-item short form of the Portrait Value Questionnaire (PVQ) (Schwartz, 2003) was used to measure *personal values*, whereas *perceived values of the contexts* were measured by an adapted version of the same questionnaire (Schwartz et al., 2006), based on which we calculated *value similarity* scores for each of the targeted five social contexts (see the *Analyses* section). For each of nine values, one item was used; Universalism was measured using two items. The PVQ presents the description of a person who cherishes the value at stake and then asks respondents to rate how similar the described person is to them on a scale ranging from “1 = very much like me” to “6 = not like me at all.” The adapted version follows the same logic but instead of asking respondents to rate how similar the described person is to them, we asked how similar the described person is to the members of the given social contexts.

As the instrument to measure *subjective well-being*, we used Diener et al.’s (1985) *Satisfaction With Life Scale*. This scale consists of five items (sample item: “In most ways, my life is close to my ideal.”) that had to be answered on a seven-point rating scale ranging from “1 = strongly disagree” to “7 = strongly agree.” Its internal consistency was α = 0.846.

**FIGURE 4:**
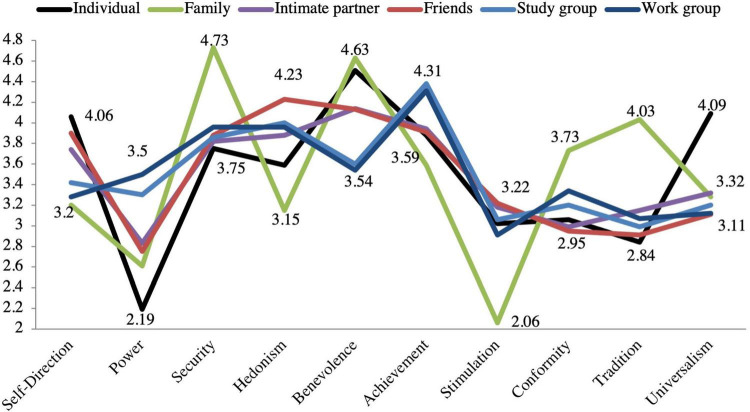
The general importance of the social contexts item.

*Context importance* was assessed in the following two ways: (1) by assessing the general importance of contexts and (2) by assessing the specific importance of contexts, namely, the relevance of the contexts to the self, dubbed identity centrality. To get an idea of the general importance of the given contexts, we allowed participants to adjust the sizes of five circles representing given social contexts that appeared on their screen from the smallest (1/5) to the biggest (5/5) to indicate the importance of each of the context. By doing this, participants’ answers are anchored relative to their other answers (Figure 4). General *context importance*, thus, even though the contexts are all presented on one screen, is still an independent measure of the importance of each of the five contexts.

The second—more specific—indicator of context importance was an *identity centrality* three-item scale used for each of the social contexts under scrutiny, as proposed by Daniel et al. (2016), originally from the Identification Questionnaire (Roccas et al., 2008). All three items referred to the importance of belonging to a given social context for the participant’s sense of identity (i.e., “Being a member of my family is an important part of my identity,” “It is important to me that I see myself as part of my family,” and “It is important to me that others see me as part of my family”). Ratings of identity centrality are fully independent of ratings for the other contexts and are being asked for on separate screens. The items had to be answered on a 6-response scale ranging from “1 = do not agree at all” to “6 = completely agree.” Cronbach’s alpha for the three-item scales ranged from α = 0.855 to α = 0.985 for the five contexts addressed.

### Analyses

Scores for the value similarity between participants’ values and each of their five social contexts’ values were calculated as Spearman’s rank (ρ) correlation coefficients between participants’ value ratings and perceived value ratings of their contexts across ten values. The procedure consisted of the following steps: for both participants’ and each of their context’s values, raw mean scores per value were corrected by subtracting the so-called MRAT (the average of ratings across all 11 items) and adding k (a constant of 3.5 based on the response scale scoring). The new pseudo scores were ranked across the ten values, and for each value, the squared distances *d* between individual’s and the context’s value rank scores were calculated. Finally, the classical Spearman formula 1–(6 × Σd_i_^2^/n^3^-n) (Hollander et al., 2014, Formula 8.64) was used to obtain the congruence coefficients between an individual and a given context (one score for each of the five social contexts). Thus, Spearman’s ρ coefficients were calculated on ipsatized scores as proposed by Schwartz.^2^ Theoretically, similarity scores can, thus, range from −1 (total misfit) to +1 (total fit). To predict subjective well-being based on value similarity with each of the contexts, moderated by each context’s identity centrality and general importance, structural equation modeling was utilized. It is worth mentioning that even though the profile correlation approach as described and used here is highly prevalent in the literature, other approaches such as polynomial regressions and response surface analyses could have been used (Wolf et al., 2020) and might well be used in further research to corroborate the findings reported below.

## Results

For illustrative purposes, we first calculated correlations between participants’ individual value preferences and subjective well-being. Pearson’s correlations with life satisfaction were significant for two out of ten values only, and—although not unusual—even those were found to be quite low. Power correlated negatively (*r* = -0.12, *n* = 997, *p* < 0.001), whereas tradition (*r* = 0.15, *n* = 997, *p* < 0.001) correlated positively.

**FIGURE 5:**
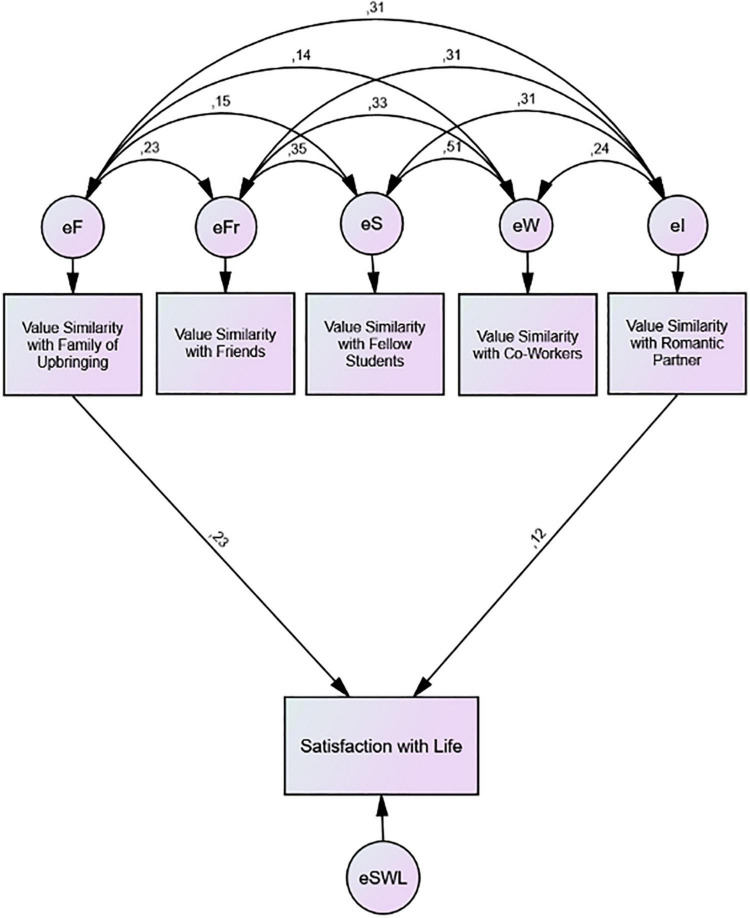
Value similarity and well-being across contexts.

Descriptive statistics for each of the 10 values reveal that Serbian emerging adults report benevolence, universalism, and self-direction as their most highly preferred values (refer to Supplementary Appendix and Table 1). At the same time, value preferences participants ascribe to their important others indicate that family is on average seen as oriented toward security, benevolence, and tradition, and friends are believed to find hedonism and benevolence most important. Study context, similarly to work context, is believed to prefer achievement values, while the intimate partner, on average, is seen as most of all valuing benevolence (refer to Supplementary Appendix and Table 2). Figure 5 presents an illustrative overview of it.

**TABLE 1:** Means, standard deviations, and correlations with confidence intervals of participants’ individual values (two-tailed).

Variable	M	SD	1	2	3	4	5	6	7	8	9
1. Self-Direction	4.06	1.26									
2. Power	2.19	1.22	−0.05 (−0.14, 0.05)								
3. Security	3.75	1.30	−0.26** (−0.35, −0.17)	−0.04 (−15, 0.05)							
4. Hedonism	3.59	1.16	−0.05 (−0.14, 0.03)	0.19** (0.09, 0.29)	−0.12** (−0.22, −0.02)						
5. Benevolence	4.51	1.06	0.04 (−0.06, 0.13)	−0.42** (−0.5, −0.32)	−0.11* (−0.2, −0.03)	−0.23** (−0.31, −0.15)					
6. Achievement	3.88	1.15	−0.14** (−0.23, −0.05)	0.16** (0.08, 0.26)	−0.08 (−0.18, 0.02)	−0.07 (−0.16, 0.03)	−0.19** (−0.27, −0.10)				
7. Stimulation	3.02	1.40	0.03 (−0.07, 0.12)	−0.04 (−0.13, 0.06)	−0.51** (−0.58, −0.42)	0.16** (0.05, 0.26)	−0.03 (−0.12, 0.07)	−0.07 (−0.16, 0.04)			
8. Conformity	3.06	1.46	−0.37** (−0.44, −0.29)	−0.08** (−0.17, 0.01)	0.19** (0.10, 0.29)	−0.3** (−0.38, −0.21)	−0.2** (−0.29, −0.11)	0.01 (−0.09, 0.11)	−0.34** (−0.42, −0.27)		
9. Tradition	2.84	1.51	−0.15** (−0.24, −0.05)	−0.37** (−0.44, −0.29)	−0.05 (−0.14, 0.06)	−0.31** (−0.38, -0.23)	0.04 (−0.05, 0.13)	−0.29** (−0.37, −0.2)	−0.16** (−0.26, −0.07)	0.02 −0.07, 0.12)	
10. Universalism	4.09	0.95	0.07 (0.03, 0.16)	−0.38** (−0.46, −0.29)	−0.07 (−0.15, 0.03)	−0.2** (−0.29, −0.11)	0.33** (0.24, 0.42)	−0.3** (−0.39, −0.2)	−0.06 (−0.16, 0.03)	−0.17 (−0.26, −0.07)	0.04 (−0.05, 0.14)

Note: *p< .05; **p < .01.

**TABLE 2:** Means and standard deviations of value types of the social contexts subjectively reported by participants.

Context	Family		Friends		Study group		Work group		Partner	
Variable	M	SD	M	SD	M	SD	M	SD	M	SD
1. Self-Direction	3.20	1.19	3.90	1.07	3.42	1.23	3.28	1.25	3.74	1.30
2. Power	2.61	1.29	2.75	1.31	3.30	1.36	3.50	1.30	2.83	1.43
3. Security	4.73	1.03	3.88	1.18	3.86	1.08	3.96	1.13	3.82	1.25
4. Hedonism	3.15	1.14	4.23	0.94	4.00	1.06	3.96	1.05	3.88	1.13
5. Benevolence	4.63	1.04	4.13	1.03	3.6	1.03	3.54	1.06	4.14	1.04
6. Achievement	3.59	1.08	3.91	1.00	4.38	1.04	4.31	1.02	3.94	1.03
7. Stimulation	2.06	1.04	3.22	1.22	3.06	1.11	2.91	1.07	3.18	1.30
8. Conformity	3.73	1.3	2.95	1.21	3.20	1.12	3.34	1.13	2.99	1.27
9. Tradition	4.03	1.23	2.91	1.26	2.99	1.07	3.07	1.11	3.15	1.30
10. Universalism	3.28	1.02	3.11	1.02	3.20	0.92	3.12	0.96	3.32	1.00

**FIGURE 6:**
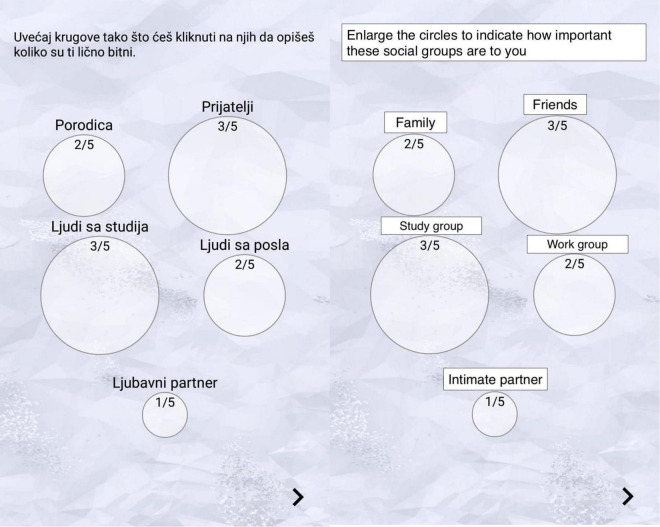
Response format used by the app (in Serbian and in its English translation).

When we calculated the overall similarity between value profiles of participants and their significant others from five contexts, we found normal distributions; across the five contexts skewness ranged from -0.317 to -0.546 with a standard error of 0.112 for each context, whereas kurtosis ranged from -0.282 to -0.660 with a standard error of 0.223 for each context. As we can see from Table 3, the highest value similarity means together with the lowest standard deviation is perceived for the intimate partner, then for friends, and then for family, while study and work contexts have the lowest similarity scores. Once we look into value similarity coefficients as correlated with satisfaction with life, we see that essentially all of them correlate positively, as we expected (please refer to the bottom row of Table 3). As correlations among predictors were low to medium in size but significant and consistent, the issue of possible multicollinearity, together with multiple moderators, led us to use structural equation modeling in AMOS (Arbuckle, 2019) for further analyses.

**TABLE 3:** Means, standard deviations, and correlations with their confidence intervals.

Variable	M	SD	1	2	3	4	5
1. Perceived value similarity with family	0.34	0.36					
2. Perceived value similarity with friends	0.36	0.35	0.23** (0.13, 0.32)				
3. Perceived value similarity with study colleagues	0.25	0.37	0.15** (0.04, 0.25)	0.35** (0.25, 0.43)			
4. Perceived value similarity with work colleagues	0.22	0.38	0.14** (0.04, 0.25)	0.33** (0.23, 0.41)	0.51** (0.42, 0.58)		
5. Perceived value similarity with intimate partner	0.41	0.32	0.31** (0.22, 0.40)	0.31** (0.23, 0.4)	0.31** (0.22, 0.38)	0.24** (0.16, 0.32)	
6. Satisfaction with life	4.54	1.27	0.27** (0.18, 0.35)	0.16** (0.06, 0.25)	0.07 (−0.02, 0.16)	0.12** (0.03, 0.20)	0.19** (0.10, 0.28)

Note: *p < .05; **p < .01.

Including all predictors in our model—*including age, gender, and their interaction as controls*—transforms our model into a saturated model, so no goodness-of-fit needs to be reported. After excluding all non-significant (*p* > *0.05)* paths, the model fit could be assessed: Significant positive contributions to well-being were found to come exclusively from value similarity coefficients with family and intimate partner (Figure 5). For none of the three controls, any significant paths (*p* < *0.05)* were found either with the five predictors or with satisfaction with life.


$$\chi^{2}=4.8,df=3,p=0.187,RMSEA=0.035,CFI=0.995$$

The findings refute our first hypothesis that the perceived value similarity of an individual with their respective social contexts will contribute to subjective well-being, regardless of the type of social context. We find the hypothesized effect in the cases of family and intimate partner contexts, but *not* for friends, studies, and work contexts. Furthermore, the second hypothesis is only partially confirmed since value similarity with friends did not contribute positively, but value similarity with family and intimate partners did.

Next, to test our third hypothesis, we included the assumed moderators, one after another, into the structural equation model—*identity centrality*, i.e., the subjective importance of a given social context for the study participants’ identities and the *general overall importance* of each context.

To include our moderators in the path model, we—separately for each moderator—performed a multigroup analysis in AMOS. We performed a mean split and split samples into two groups (low and high) for identity centrality of family and identity centrality of the intimate partner, as the only two contexts contributing to the outcome. For identity centrality, there were no differences between the groups (χ^2^ for family increased from 5.97, *df* = 6, *p* = 0.427 in the unconstrained to 7.12, *df* = 7, *p* = 0.416 in the constrained model and from 11.01, *df* = 6, *p* = 0.088 to 11.06, *df* = 7, *p* = 0.136 for the intimate partner). In both cases, Δ_χ_^2^ is not significant with one degree of freedom. Identity centrality, thus, was *not* found to be a moderator of any of the investigated relationships. When it comes to the general importance of groups, there was no significant difference when comparing groups differing in the importance of family (in the unconstrained model χ^2^ = 5.97, *df* = 6, *p* = 0.426 in the constrained model χ^2^ = 6.92, *df* = 7, *p* = 0.438). Attributing low vs. high importance to the intimate partnership *did make a significant difference* as to whether value similarity was positively related to well-being. The difference in the importance of the intimate partner was found to be significant (in the unconstrained model χ^2^ = 5.67, *df* = 6, *p* = 0.461 in the constrained χ^2^ = 14.37, *df* = 7, *p* = 0.045). The Δ_χ_^2^ of 8.70 is significant at *p* = 0.003. Value similarity with one’s intimate partner predicts satisfaction with life if the intimate partner is *not* so important, which is *not* in accordance with our third hypothesis.

## Discussion

In this study, we looked into similarities between the values of emerging adults and values they perceive as being held by important others (family, friends, study and work colleagues, and intimate partners). The study aimed to investigate the relationship between value similarity and subjective well-being among Serbian emerging adults, as assumed and confirmed in previous studies in other populations and with fewer social contexts. Using a short form of Schwartz’s value questionnaire to calculate perceived value similarity with five different social contexts consisting of important others and relating these similarity scores with Diener’s life satisfaction scores, we obtained the following results:

First, only looking at correlations, value similarity with immediate social contexts contributes to participants’ subjective well-being. Having values similar to perceived values of each social context except for that of study colleagues is significantly positively correlated with satisfaction with life. This is very much in line with the previous studies (e.g., Knafo and Assor, 2007; Morley, 2007; Daddis, 2008), which in addition typically also ascertained a positive association between value similarity and well-being also for the study colleague case (e.g., Sagiv and Schwartz, 2000; Musiol and Boehnke, 2013). However, structural equation models revealed that only value similarity with family and intimate partner contributed to the well-being of the study participants. There are different ways to interpret this result. One interpretation could be that these contexts are primary intimacy groups in comparison to study and work groups, which are considered to be task groups as hypothesized. However, if we follow this reasoning, it is surprising not to see a meaningful association of value similarity with friends and satisfaction with life as well. It seems puzzling at first glance that value similarity with one’s study context emerges as unrelated to subjective well-being. Readers should, however, bear in mind that in an Eastern European country such as Serbia, the choice of study major is often less of a personal choice aligned with individual interests, personality traits, and values, but more a utilitarian choice of a path toward a more financially secure career (Tomanović and Ignjatović, 2006). Furthermore, another difference between previously mentioned studies and the current one is that here values of the contexts were included as *perceived* by respondents, whereas in other studies value ratings often came from “the others” themselves. This provides an interesting turn for potential future studies since interactions being bidirectional, value similarity defined as such could also be seen as a relationship quality indicator (as proposed in Hoellger et al., 2021a; Hoellger et al., 2021b).

Obviously, separately relating value similarity with life satisfaction in five different contexts are affected by a certain degree of overlap: Value similarity with others is likely to be related to life satisfaction regardless of the exact group of others under scrutiny. It was, thus, necessary to also test the question of a relationship between value similarity and subjective well-being multivariately—in a structural equation model. Results of that analysis showed that only value similarity with family (of upbringing) and with one’s intimate partner (potential future family) predicted well-being significantly. This finding challenges results from previous studies that looked at specific social contexts separately (e.g., Morley, 2007; Pereyra-Rojas et al., 2017).

Significant others, such as one’s family and intimate partner, are social contexts of particularly close and long-term nature, and, in line with our overall thinking, they were the only ones to contribute to subjective well-being. Value similarity with friends, even though perceived in the literature as important, did not play a role, which might best be explained as follows. First, a recent study suggests that to eventually achieve adulthood, emerging adults shift their focus from friends toward their intimate partners (Barry et al., 2009). Second, friends as a social context no longer encompass only one group of people; rather, emerging adults interact with multiple social groups both online and offline (e.g., Vannucci et al., 2017). The implications of this are twofold: It might be that the task given to our participants to the describe values of their friends is to some degree ambiguous since it could refer to a very inhomogeneous group of people from diverse social contexts. Since our approach is rather novel and the complexity is already quite high with five different social contexts, we refrained from accounting for group size (intimate partner almost always refers to only one person, whereas all other contexts potentially encompass many more members). Not accounting for group size obviously is a limitation of this study, since when asked about contexts with multiple people, respondents may be giving differently focused answers (e.g., based on the typical member of the group, based on the most dominant one, or the most relevant based on diverse criteria). This is something future research should be accounted for, possibly in a form of more precise instructions when asking for the values of groups. In addition, since friendships are voluntary relationships (Reis et al., 2000), they depend on the long-term commitment which is not characteristic of this population due to an abundance of alternative friend groups and thus can be less long-lasting and less relevant value-wise (Collins and van Dulmen, 2006). Finally, long-lasting partners are typically chosen based on homogamy for meaning sharing and eventual transmission of values purposes (Blackwell and Lichter, 2004), which might not be the basis for the choice of friends or friendship-based social contexts.

Finally, interestingly enough, identity centrality, measured in a “classic” way *via* a highly reliable three-item scale, did not emerge as a moderator either for family or for an intimate partner. It is irrelevant whether one’s family of upbringing or one’s intimate partner is central to one’s identity: Perceived value similarity with them always has a positive effect on well-being. However, if one assesses the general importance of these two social contexts, one astounding finding arises. Although there is also no moderation effect for family, the significant moderation effect found for value similarity with an intimate partner is surprising but telling: if my intimate partner is more important to me, the similarity of our values is significantly less positively related to well-being than if my partner is less important. In the latter case, i.e., when my partner is not so important to me, value similarity becomes more important for well-being. One is inclined to speculate that if participants of the study do not plan on forming a long-lasting partnership with their current intimate partner, having the same values as the current partner becomes *more* important to their well-being possibly because they are being negotiated about, thus actively enhancing or decreasing the well-being. Taken to the extreme, one could maybe even say: Where there is no love, value similarity is the primary fountain of well-being. In contrast, one way to explain value similarity as having less impact on an individual’s subjective well-being when the importance of the partner is high is the shift of focus to other personality characteristics (Keizer and Komter, 2015), daily interactions (Chi et al., 2013), or even circumstances—devotedness to the relationship connected to the achievement of the adult age might play a role *via* emotional support and companionship.

In summary, this study tested the person–environment value fit hypothesis in a multiple-context setting, which is ecologically more valid compared to the singular settings studied in much of the previous research. Out of value similarity with important others from five immediate contexts, similarity with family and an intimate partner were the only ones that significantly contributed to life satisfaction. However, our hypothesis about the effect of the identity centrality moderator was essentially disconfirmed. In the future, other potential moderators need to be investigated, such as the affordances social contexts provide and social sanctions they (threaten to) impose. This is important to get a better grasp at what is it about the context importance, if not identity centrality, that makes value similarity less or more relevant to subjective well-being.

Obviously, the present research has certain limitations. Like many studies in psychology, the sample drawn for the study was haphazard and not a random probability sample from a specified population. Second, the population from which the sample was drawn was made up exclusively of Serbian emerging adults, which leads to the question to which degree results can be generalized beyond Serbia. However, aside from the fact that we have no intentions of generalizing information about specific contexts that could vary cross-culturally, the phenomenon is considered developmental in its nature and thus universal to emerging adults, at least in the WEIRD^3^ world. Thirdly, we need to acknowledge the fact that the social contexts were given and not freely elicited by participants, and thus may not have been equally ecologically valid for every study participant. That issue was addressed by offering participants the choice to exclude one or more of the presented contexts if not applicable or relevant and add a new one to indicate if there is information missing for the individual participant. However, participants who made use of this option are not included in the analyses reported here because including them would have thrust a non-negligible issue of how to handle missing data on the performed analyses. Implicitly one could argue that the subsample studied here is a socially well-integrated group of people who “have” all five social contexts, whereas similarly sized other groups of study participants indicated that they do not “have” all contexts. This means that the generalizability of our findings is reduced to well-integrated emerging adults from Serbia.

Furthermore, the repetitive nature of the questionnaire could have induced automatic responses due to overburdening. In collaboration with a programmer, we put our effort into designing a user-friendly, interactive, and idiosyncratic interface, which we believe at least reduced the occurrence of boredom and automatic responses and the feedback was, in fact, very positive. Finally, the size of the relationship between value similarity and well-being being not more than moderate is rather in line with other studies in the field (e.g., Sagiv and Schwartz, 2000).

Nevertheless, this study contributes to the existing theoretical knowledge about the relationship between personal values and subjective well-being. This is accomplished by not only applying but also expanding on the person–environment fit in a new, ecologically externally valid manner by including multiple social contexts for the first time. Yet, it seems that only significant others matter out of all of the important others from multiple social contexts included.

## Data Availability Statement

The raw data supporting the conclusions of this article will be made available by the authors upon request, without undue reservation.

## Ethics Statement

The studies involving human participants were reviewed and approved by Bremen International Graduate School of Social Sciences Ethics Committee. The participants provided their written informed consent to participate in this study.

## Author Contributions

JB, MB, and KB contributed to the conception and design of the study. JB designed the data collection instruments and collected the data. JB and MB wrote the first draft of the manuscript. JB and KB performed the statistical analyses. All authors contributed to the article, edited the article, and approved the submitted version.

## Conflict of Interest

The authors declare that the research was conducted in the absence of any commercial or financial relationships that could be construed as a potential conflict of interest.

## Publisher’s Note

All claims expressed in this article are solely those of the authors and do not necessarily represent those of their affiliated organizations, or those of the publisher, the editors and the reviewers. Any product that may be evaluated in this article, or claim that may be made by its manufacturer, is not guaranteed or endorsed by the publisher.

## Footnotes

1 For Schwartz’s refined model of personal values, please refer to Schwartz et al. (2012).

2
http://essedunet.nsd.uib.no/cms/topics/1/4/4.html

3 Western Educated Industrialized Rich Democratic.
