# Supplementary material for: Perceived Value Similarity With Important Others: Well-Being Implications for Emerging Adults
Source: Front Psychol. 2022 May 30;13:716952. doi: 10.3389/fpsyg.2022.716952 (PMC9190204; doi:10.3389/fpsyg.2022.716952)
Supplement: Supplementary file 1 [file Data_Sheet_1.PDF]

J:\Orga\SHSS\Workgroups\Workgroup Boehnke\Faculty02\Eigene Dateien\BIGSSS\tested model.amw

Analysis Summary

Date and Time

Date: Donnerstag, 7. Oktober 2021
Time: 17:17:19

Title

tested model: Donnerstag, 7. Oktober 2021 17:17

Groups

Group number 1 (Group number 1)

Notes for Group (Group number 1)

The model is recursive.
Sample size = 479

Variable Summary (Group number 1)

Your model contains the following variables (Group number 1)

Observed, endogenous variables

SWL
rGIVandFAMV
rGIVandFRIV
rGIVandEDUV
rGIVandWORKV
rGIVandLOVEV

Unobserved, exogenous variables

sSAT
eF
eFr
eS
eW
eI

Variable counts (Group number 1)

Number of variables in your model: 12
Number of observed variables: 6
Number of unobserved variables: 6
Number of exogenous variables: 6
Number of endogenous variables: 6

Parameter Summary (Group number 1)

	Weights	Covariances	Variances	Means	Intercepts	Total
**Fixed**	6	0	0	6	0	12
**Labeled**	0	0	0	0	0	0
**Unlabeled**	2	10	6	0	6	24
**Total**	8	10	6	6	6	36

Models

Default model (Default model)

Notes for Model (Default model)

Computation of degrees of freedom (Default model)

Number of distinct sample moments: 27
Number of distinct parameters to be estimated: 24
Degrees of freedom (27 - 24): 3

Result (Default model)

Minimum was achieved
Chi-square = 4,799
Degrees of freedom = 3
Probability level = ,187

Group number 1 (Group number 1 - Default model)

Estimates (Group number 1 - Default model)

Scalar Estimates (Group number 1 - Default model)

Maximum Likelihood Estimates

Regression Weights: (Group number 1 - Default model)

			Estimate	S.E.	C.R.	P	Label
SWL	<---	rGIVandFAMV	,826	,162	5,084	***	
SWL	<---	rGIVandLOVEV	,471	,182	2,584	,010	

Standardized Regression Weights: (Group number 1 - Default model)

			Estimate
SWL	<---	rGIVandFAMV	,234
SWL	<---	rGIVandLOVEV	,119

Intercepts: (Group number 1 - Default model)

	Estimate	S.E.	C.R.	P	Label
**rGIVandFAMV**	,343	,016	20,943	***	
**rGIVandLOVEV**	,406	,015	27,750	***	
**SWL**	4,068	,095	42,690	***	
**rGIVandFRIV**	,362	,016	22,950	***	
**rGIVandEDUV**	,250	,017	14,849	***	
**rGIVandWORKV**	,224	,017	13,018	***	

Covariances: (Group number 1 - Default model)

			Estimate	S.E.	C.R.	P	Label
eF	<-->	eI	,036	,005	6,495	***	
eFr	<-->	eF	,028	,006	4,802	***	
eS	<-->	eF	,019	,006	3,176	,001	
eW	<-->	eF	,019	,006	2,999	,003	
eFr	<-->	eS	,044	,006	7,229	***	
eS	<-->	eW	,070	,007	9,865	***	
eS	<-->	eI	,036	,006	6,398	***	
eW	<-->	eI	,029	,006	5,155	***	
eFr	<-->	eI	,035	,005	6,527	***	
eFr	<-->	eW	,042	,006	6,796	***	

Correlations: (Group number 1 - Default model)

			Estimate
eF	<-->	eI	,311
eFr	<-->	eF	,225
eS	<-->	eF	,147
eW	<-->	eF	,138
eFr	<-->	eS	,350
eS	<-->	eW	,506
eS	<-->	eI	,306
eW	<-->	eI	,243
eFr	<-->	eI	,313
eFr	<-->	eW	,327

Variances: (Group number 1 - Default model)

	Estimate	S.E.	C.R.	P	Label
**eF**	,129	,008	15,460	***	
**eI**	,102	,007	15,460	***	
**sSAT**	1,466	,095	15,460	***	
**eFr**	,119	,008	15,460	***	
**eS**	,135	,009	15,460	***	
**eW**	,142	,009	15,460	***	

Minimization History (Default model)

Iteration		Negative eigenvalues	Condition #	Smallest eigenvalue	Diameter	F	NTries	Ratio
0	e	1		-,017	9999,000	394,458	0	9999,000
1	e	0	48,894		,548	88,955	18	,944
2	e	0	57,690		,315	23,190	1	1,125
3	e	0	71,865		,252	6,633	1	1,173
4	e	0	94,315		,119	4,837	1	1,089
5	e	0	99,134		,021	4,799	1	1,016
6	e	0	98,747		,001	4,799	1	1,000

Model Fit Summary

CMIN

Model	NPAR	CMIN	DF	P	CMIN/DF
**Default model**	24	4,799	3	,187	1,600
**Saturated model**	27	,000	0		
**Independence model**	12	402,432	15	,000	26,829

Baseline Comparisons

Model	NFI Delta1	RFI rho1	IFI Delta2	TLI rho2	CFI
**Default model**	,988	,940	,995	,977	,995
**Saturated model**	1,000		1,000		1,000
**Independence model**	,000	,000	,000	,000	,000

Parsimony-Adjusted Measures

Model	PRATIO	PNFI	PCFI
**Default model**	,200	,198	,199
**Saturated model**	,000	,000	,000
**Independence model**	1,000	,000	,000

NCP

Model	NCP	LO 90	HI 90
Default model	1,799	,000	12,017
Saturated model	,000	,000	,000
Independence model	387,432	325,722	456,563

FMIN

Model	FMIN	F0	LO 90	HI 90
Default model	,010	,004	,000	,025
Saturated model	,000	,000	,000	,000
Independence model	,842	,811	,681	,955

RMSEA

Model	RMSEA	LO 90	HI 90	PCLOSE
Default model	,035	,000	,092	,585
Independence model	,232	,213	,252	,000

AIC

Model	AIC	BCC	BIC	CAIC
Default model	52,799	53,512		
Saturated model	54,000	54,803		
Independence model	426,432	426,788		

ECVI

Model	ECVI	LO 90	HI 90	MECVI
Default model	,110	,107	,132	,112
Saturated model	,113	,113	,113	,115
Independence model	,892	,763	1,037	,893

HOELTER

Model	HOELTER .05	HOELTER .01
Default model	779	1131
Independence model	30	37

Execution time summary

Minimization: ,018
Miscellaneous: 2,563
Bootstrap: ,000

Total: 2,581
